# Betel nut and tobacco chewing; potential risk factors of cancer of oesophagus in Assam, India

**DOI:** 10.1054/bjoc.2001.1920

**Published:** 2001-09

**Authors:** R K Phukan, M S Ali, C K Chetia, J Mahanta

**Affiliations:** Regional Medical Research Centre, Indian Council of Medical Research, North East Region, Assam, Dibrugarh, India; Hospital Tumour Registry, Indian Council of Medical Research, Assam Medical College, Dibrugarh, Assam, India; Department of Statistics, Dibrugarh University, Dibrugarh, Assam, India

**Keywords:** oesophageal cancer, betel nut chewing, tobacco chewing, Assam

## Abstract

Cancer of the oesophagus is the most commonly diagnosed cancer in males in Assam, in north-eastern India, and ranks second for females. The chewing of betel nut, with or without tobacco and prepared in various ways, is a common practice in the region and a case–control study has been designed to study the pattern of risk associated with different ways of preparing and chewing the nuts. 358 newly diagnosed male patients and 144 female have been interviewed together with 2 control subjects for each case chosen at random from among the attendants who accompanied patients to hospital. There were significant trends in risk ratios associated with the frequency of chewing each day, with the duration of chewing in years and with the age at which the habit was started that were apparent for both males and females and which remained significant after allowance was made for other known risk factors, notably tobacco smoking and alcohol consumption. The adjusted ratios, in comparison with non-chewers, were 13.3 M and 5.7 F for chewing more than 20 times a day, 10.6 M and 7.2 F for persons who had chewed for more than 20 years and 10.3 M and 5.3 F for those who had started before the age of 20. Among the different combinations of ingredients that were chewed the adjusted odds ratios were highest for those who had been using fermented betel nut with any form of tobacco (7.1 M and 3.6 F). The risk associated with tobacco smoking and alcohol consumption, which are high in some parts of the world, were less in Assam than those associated with the chewing of betel nut. © 2001 Cancer Research Campaign http://www.bjcancer.com


*British Journal of Cancer* (2001) **85**(5), 661–667
© 2001 Cancer Research Campaign
doi: 10.1054/ bjoc.2001.1920, available online at http://www.idealibrary.com on IDEAL

http://www.bjcancer.com

### Betel nut and tobacco chewing; potential risk factors of cancer of oesophagus in Assam, India

**RK Phukan[1], MS Ali[2], CK Chetia[3] and J Mahanta**

[1]Regional Medical Research Centre, Indian Council of Medical Research, Dibrugarh, North East Region, Assam, India; [2]Hospital Tumour Registry, Indian Council of Medical Research, Assam Medical College, Dibrugarh, Assam, India; [3]Department of Statistics, Dibrugarh University, Dibrugarh, Assam, India

**Summary** Cancer of the oesophagus is the most commonly diagnosed cancer in males in Assam, in north-eastern India, and ranks second for females. The chewing of betel nut, with or without tobacco and prepared in various ways, is a common practice in the region and a case–control study has been designed to study the pattern of risk associated with different ways of preparing and chewing the nuts. 358 newly diagnosed male patients and 144 female have been interviewed together with 2 control subjects for each case chosen at random from among the attendants who accompanied patients to hospital. There were significant trends in risk ratios associated with the frequency of chewing each day, with the duration of chewing in years and with the age at which the habit was started that were apparent for both males and females and which remained significant after allowance was made for other known risk factors, notably tobacco smoking and alcohol consumption. The adjusted ratios, in comparison with non-chewers, were 13.3 M and 5.7 F for chewing more than 20 times a day, 10.6 M and 7.2 F for persons who had chewed for more than 20 years and 10.3 M and 5.3 F for those who had started before the age of 20. Among the different combinations of ingredients that were chewed the adjusted odds ratios were highest for those who had been using fermented betel nut with any form of tobacco (7.1 M and 3.6 F). The risk associated with tobacco smoking and alcohol consumption, which are high in some parts of the world, were less in Assam than those associated with the chewing of betel nut. © 2001 Cancer Research Campaign http://www.bjcancer.com

**Keywords**: oesophageal cancer; betel nut chewing; tobacco chewing; Assam

Cancer data, from both population-based and hospital-based cancer registries in India, showed the highest incidence of oesophageal cancer to occur in Assam in the north east of the country, followed by Bangalore and Bombay (NCRP, 1984–1989). In Western populations, oesophageal cancer (especially amongst men) seems to be mostly due to a combination of tobacco smoking and alcohol consumption (Tuyns et al, 1977). Poor nutrition may increase susceptibility in many parts of the world and various local factors such as very hot liquids, and the consumption of pyrolysed products such as opium dross in Iran or dottle from the stem of tobacco pipes in South Africa seem to compound the risk and to produce very high rates even in areas where tobacco smoking and alcohol consumption are rare (Munos and Day, 1997; Kinjo et al, 1998). Aetiological studies in India have quantified the risks of oesophageal cancer associated with betel nut chewing and the consumption of alcohol and tobacco in Bombay and Bangalore (Jussawalla, 1971; Jussawalla and Deshpande, 1971; Nandakumar et al, 1996) but no such investigation has been made in Assam where certain ingredients and methods of preparetion of the betel nut quid differ from those common in other parts of India.

In Assam 'raw' ('green'), 'ripe' ('red') and 'fermented' ('underground', 'processed') betel nuts are all chewed. The latter, known locally as 'Bura Tamul', is prepared in a 4–5 foot hole in the ground where ripe betel nuts are left for 3–4 months covered with bark from the betel tree, cow dung and soil. During the period of fermentation the outer fibrous shell of the nuts decays. Chopped or crushed nuts at the different stages of ripening or decay are wrapped in betel leaf and are chewed with or without tobacco. 'Dhapat', dried tobacco leaf that may be treated with lime (calcium oxide), is sometimes added to the betel nut in the quid while a mixture of finely cut and dried, 'raw' or 'ripe' betel nut ('Supari') and finely cut, scented tobacco ('Zarda') is also chewed. In Assam a larger proportion of betel nut is included in the quid and fewer leaves than in the 'pan' which is chewed in Bombay and which includes only a very small quantity of betel nut that is always processed ('fermented'). As in Assam, the Bombay quid may also include tobacco. Dried tobacco chewed alone in Assam is known locally as 'Chadha'. Whatever the composition of the quids, they are usually retained in the mouth for about 20 to 25 minutes but occasionally the mixture may be retained in the mandibular groove during sleep (Bhansle et al, 1979).

A case–control study has been carried out in collaboration with the Dr. Bhubaneswar Barooah Cancer Institute (BBCI) in Guwahati (the largest city in Assam) to investigate the risks associated with the various chewing habits that are practised in the state and to estimate the effect independently of tobacco and alcohol consumption.

### METHODS

The BBCI is one of the regional cancer treatment and research centres in India and provides treatment for patients from the 7 north-eastern states, of which Assam is the largest, (total population 31.4 million (1991 Census)). The study was conducted from July 1997 to June 1998 during which period 3720 cases of all types of cancer were registered and 590 new cancer of the oesophagus cases. All suspected cases of cancer of the oesophagus were directed to the social investigator(s) of the project for interview

*Received 16 November 2000*
*Revised 27 April 2001*
*Accepted 27 April 2001*

*Correspondence to:* J Mahanta before referral to the medical consultant. At the same time information was collected from the attendants who accompanied cancer patients and who provided a readily available and cooperative source of controls from the same socio-economic background as the patients. A final group of matched controls (2 for each patient) were selected by random pairing of the cases with subjects from the pool of controls after matching for sex and age (within ± 5 years).

Only cases confirmed by microscopy and for whom the oesophagus was the primary site of cancer were included in the study. Out of the total cases 93.2% had squamous cell carcinoma, 5.2% had adenocarcinoma and 1.6% other types of cancers. Patients with advanced disease (20), where the tumour had spread so as to obscure the primary site, patients with recurrent cancer (20) and those who were too elderly (12) and who refused to be interviewed (31) were excluded from the study. A total of 502 patients were finally included (358 men and 144 women).

Details of age and sex and various demographic variables were collected in the course of the interviews as well as details of personal habits that included tobacco smoking and the consumption of alcohol as well as chewing practices. A pre-designed, pretested questionnaire was designed specifically for the study. The selection of controls from among the persons bringing the patients to hospital is likely to have minimised differences of socioeconomic conditions and also of adequacy of nutrition between the patients and controls and these have not been investigated further.

Analysis of the data was by multiple logistic regression (Breslow and Day, 1980) from which ratios of relative risk (odds ratio = exp(β)) and standard errors were derived for betel nut chewing, tobacco smoking and alcohol consumption (with or without stratified adjustment of each factor for the other 2, potentially confounding, habits). In the multifactorial models, the ‘other’ factors were fitted before the exposure factor of interest.

Estimation of the proportion of cases of a disease attributable to exposure to a particular factor has been done by calculating the ‘aetiological fractions’ for each variable (Levin, 1953).

### RESULTS

The adjusted risks associated with the chewing of betel nut were higher than those for tobacco smoking and alcohol consumption at all levels of consumption (Tables 1–3). However, for all 3 habits there were significantly elevated ORs at high levels of intake or after a long duration of consumption and clear indications of dose–response effects for all 3 habits. The adjusted ORs for persons who chewed more than 20 times a day in comparison with non-chewers were 13.3 for males and 8.4 for females ($P < 0.001$ for both comparisons) (Table 1) whereas the adjusted ORs for smoking more than 20 times a day were 3.7 and 2.5 ($P < 0.001$ and $P = 0.03$) (Table 2) and the adjusted ORs for the highest level of alcohol consumption were 4.8 ($P = 0.05$) for males (drinking more than 10 times a week) and 3.6 ($P = 0.006$) for females (drinking 5–10 times a week) (Table 3).

65% of men in the control population and 38% of women were chewers but only 24% of the men and 3% of the women smoked tobacco and only 24% and 4% consumed alcoholic drinks. In view of the lower population-exposure and of the lower adjusted ORs for the smoking and drinking habits, compared with those for chewing, the detailed results are tabulated (Tables 2 and 3) but are not mentioned further in the text.

**Table 1** Risk estimates of betelnut chewing habits and dose–response parameters with or without adjustment for smoking and alcohol

| Chewing | Male | | | | | Female | | | | |
|---|---|---|---|---|---|---|---|---|---|---|
| Characteristics | Ca/Co | OR (95% CI) | *P* value | Adj OR (95% CI) | *P* value | Ca/Co | OR (95% CI) | *P* value | Adj OR (95% CI) | *P* value |
| Non-chewer | 30/249 | 1 | | | | 34/153 | 1 | | | |
| Chewers | 328/457 | 5.8 (2.3–10.2) | < 0.001 | 2.6 (1.3–7.4) | 0.045 | 110/135 | 3.7 (1.6–10.3) | < 0.001 | 1.9 (0.02–7.8) | 0.062 |
| Frequency (per day) | | | | | | | | | | |
| 1–4 | 60/169 | 2.9 (1.3–8.4) | < 0.01 | 2.3 (0.2–8.4) | 0.041 | 25/60 | 1.9 (0.89–5.3) | 0.093 | 1.5 (0.07–5.7) | 0.093 |
| 5–10 | 71/170 | 3.5 (1.9–10.4) | < 0.001 | 2.5 (0.7–9.6) | 0.021 | 17/34 | 2.3 (1.02–8.4) | < 0.05 | 1.7 (0.02–6.4) | 0.072 |
| 11–20 | 80/77 | 8.6 (3.9–15.3) | < 0.001 | 4.8 (1.3–8.4) | < 0.001 | 38/25 | 6.8 (2.5–13.8) | < 0.001 | 2.3 (0.5–6.5) | 0.031 |
| 20 + | 117/51 | 19 (9.4–28.2) | < 0.001 | 13.3 (4.5–24.6) | < 0.001 | 30/16 | 8.4 (4.3–19.6) | < 0.001 | 5.7 (2.5–17.6) | < 0.001 |
| Duration (years) | | | | | | | | | | |
| <10 | 51/180 | 2.4 1.1–8.2 | < 0.05 | 1.8 (0.09–7.1) | 0.083 | 25/71 | 1.6 (1.2–6.8) | 0.087 | 1.2 (0.07–5.2) | 0.143 |
| 10–19 | 64/165 | 3.2 1.8–10.5 | < 0.001 | 1.9 (0.06–5.5) | 0.068 | 42/49 | 3.9 (1.4–8.5) | < 0.01 | 1.7 (0.03–6.1) | 0.082 |
| 20 + | 213/122 | 14.5 5.6–23.9 | < 0.001 | 10.6 (5.6–17.3) | < 0.001 | 43/15 | 12.9 (2.0–18.8) | < 0.001 | 7.2 (2.6–14.2) | < 0.001 |
| Age start (years) | | | | | | | | | | |
| <20 | 154/90 | 14.2 (5.4–26.3) | < 0.001 | 10.3 (3.1–19.7) | < 0.001 | 49/27 | 8.2 (2.5–20.8) | <0.001 | 5.3 (2.1–18.2) | < 0.001 |
| 20–29 | 142/178 | 6.6 (2.3–12.4) | < 0.001 | 4.8 (1.4–9.5) | < 0.001 | 40/30 | 6 (1.1–15.6) | <0.001 | 3.9 (1.5–7.8) | < 0.001 |
| 30+ | 32/199 | 1.3 (0.8–5.8) | 0.075 | 0.8 (0.07–4.2) | 0.371 | 21/78 | 1.2 (0.9–6.7) | 0.064 | 0.5 (0.02–6.1) | 0.561 |

Ca = cases; Co = controls; OR = odds ratio.

 *© 2001 Cancer Research Campaign*

BJOC 01-1920 661-667 20/8/01 3:06 pm Page 663

**Table 2** Risk estimates of smoking habits and dose–response parameters with or without adjustment for chewing and alcohol

| Smoking characteristics | Male | | | | | Female | | | | |
|---|---|---|---|---|---|---|---|---|---|---|
| | Ca/Co | OR (95% CI) | *P* value | Adj OR (95% CI) | *P* value | Ca/Co | OR (95% CI) | *P* value | Adj OR (95% CI) | *P* value |
| Non-smokers | 198/544 | 1 | | | | 129/278 | 1 | | | |
| Smokers | 160/172 | 2.6 (1.2–8.1) | 0.031 | 1.2 (0.03–6.5) | 0.07 | 15/10 | 3.2 (1.9–10.5) | 0.04 | 1.8 (0.05–5.8) | 0.34 |
| Frequency (per day) | | | | | | | | | | |
| 1–4 | 20/50 | 1.1 (0.05–4.5) | 0.72 | 0.85 (0.04–3.5) | 0.46 | 3//3 | 2.2 (0.9–9.4) | 0.058 | 1.6 (0.3–4.5) | 0.43 |
| 5–10 | 35/48 | 2 (0.02–5.8) | 0.31 | 1.3 (0.03–3.7) | 0.68 | 5//4 | 2.7 (1.3–10.4) | 0.052 | 1.8 (0.8–6.2) | 0.34 |
| 11–20 | 47/41 | 3.1 (1.5–8.6) | 0.006 | 2.5 (1.4–7.6) | 0.007 | 4//2 | 4.3 (1.8–15.8) | < 0.001 | 2.1 (0.6–10.3) | 0.04 |
| 20 + | 58/33 | 4.8 (2.5–12.5) | < 0.001 | 3.7 (1.8–8.5) | < 0.001 | 3//1 | 6.4 (3.6–20.5) | < 0.001 | 2.5 (0.8–8.5) | 0.03 |
| Duration (years) | | | | | | | | | | |
| <10 | 38/68 | 1.5 (0.4–6.5) | 0.65 | 0.68 (0.04–3.5) | 0.69 | 5//6 | 1.8 (0.4–4.2) | 0.48 | 0.6 (0.03–5.1) | 0.15 |
| 10–19 | 56/53 | 2.9 (0.8–8.3) | 0.07 | 1.5 (0.4–4.6) | 0.31 | 7//3 | 5 (2.6–12.2) | < 0.001 | 2.7 (0.9–10.8) | 0.03 |
| 20 + | 66/51 | 3.6 (1.4–11.5) | 0.005 | 2.8 (0.3–6.5) | 0.09 | 3//1 | 6.5 (3.2–18.3) | < 0.001 | 3.2 (1.5–9.5) | 0.007 |
| Age at start (years) | | | | | | | | | | |
| < 20 | 84/45 | 5.1 (1.4–14.50) | < 0.001 | 4.4 (1.8–16.3) | < 0.001 | 6//2 | 6.5 (2.3–14.5) | < 0.001 | 2.3 (0.6–9.2) | 0.02 |
| 20–29 | 46/56 | 2.2 (0.6–9.5) | 0.15 | 1.7 (0.7–8.5) | 0.59 | 6//3 | 4.3 (1.8–11.4) | < 0.001 | 2.1 (0.9–8.7) | 0.004 |
| 30 + | 30/71 | 1.2 (0.04–5.6) | 0.35 | 0.8 (0.03–4.5) | 0.76 | 3//5 | 1.3 (0.9–8.8) | 0.46 | 0.4 (0.07–3.9) | 0.48 |
| Type of smoking | | | | | | | | | | |
| Bidi | 72/55 | 3.6 (1.8–9.5) | 0.007 | 2.8 (1.3–7.4) | 0.76 | 7//3 | 5 (2.1–12.6) | < 0.001 | 2.4 (1.3–8.3) | 0.006 |
| Cigarette | 56/73 | 2.1 (1.3–8.6) | 0.35 | 1.5 (0.8–6.3) | 0.46 | 5//3 | 3.6 (1.4–8.9) | 0.004 | 1.8 (0.06–8.6) | 0.08 |
| Others | 32/44 | 1.9 (0.8–6.3) | 0.61 | 1.2 (0.5–7.8) | 0.58 | 3//4 | 1.6 (0.7–4.5) | 0.21 | 0.7 (0.07–6.3) | 0.43 |

Consideration of the duration of chewing habits and of the age at which the habit was taken up (Table 1) shows adjusted ORs of 10.6 and 12.9 for men and women who had been chewing for more than 20 years and of 10.3 and 5.3 for those who started the habit before the age of 20 ($P < 0.001$ in each instance).

The risks associated with the different types of quid that are chewed are shown in Table 4. The highest adjusted risks for men are associated with the chewing of betel nut together with tobacco (both Dhapat (OR 7.1, $P < 0.01$ where fermented betel nut is used and OR 3.1, $P < 0.01$ where green or red betel nut is used) and Zarda (OR 6.6, $P < 0.001$)). For men who chew tobacco alone (Chadha) the risk is also elevated (OR 4.9, $P < 0.001$). The pattern for women is similar but not identical. However, the numbers are smaller than those for men and so the ORs are likely to be less stable.

For both men and women the adjusted risks associated with the chewing of betel nut without tobacco are lower than those where tobacco is used, especially when the tobacco is added to fermented nut (OR 7.1, $P < 0.01$ for men and 3.6, $P < 0.001$ for women). The ORs associated with taking just green or red betel nut are 1.9 for males and 0.5 for females, neither differing significantly from the risk in non-chewers. For chewers of fermented betel nut without tobacco there is a slightly raised risk for males (OR 2.3, $P < 0.05$) and no elevation of risk for females (OR 0.8, $P = 0.351$).

The risks for persons who spit out the juices of the quid contrasted with those who swallow them and for those who retain the quid in the mouth for longer periods of time are given in Table 5. For males there is a clear trend in increasing risk from those who spit or swallow sometimes (adjusted ORs of 1.4 and 1.6 that are not significantly different from the risk in non-chewers) to those who both swallow the juices and retain the quid in the mouth (OR 6.3, $P < 0.001$). For women the pattern is less clear but the numbers who retain the quid in the mouth with or without swallowing are very few.

The combined effect of betel nut chewing and smoking as well as chewing and alcohol drinking are shown in Table 6 and Table 7. The highest risks for men (OR = 15.3) and women (OR = 27.4) were found to be associated when fermented betel nut was used in combination with tobacco and bidi smoking. A combination of fermented betel nut with tobacco and non-commercial alcoholic drinks showed a highly elevated risk (OR = 18.5 M and OR = 13.5 F).

The risks for persons who practice different combinations of the three habits are given in Table 8. For both men and women, the highest risks are among those who practice all three, chewing betel nut, smoking tobacco and consuming alcoholic drinks, (ORs 13.6 and 11.8); and then among those who chew and smoke (ORs 8.4 and 8.1). The ORs for chewing and drinking are also elevated but to a slightly lesser extent (ORs 5.5 and 7.6). The risks associated with the practice of just one of the habit again show chewing (ORs 3.4 for men and 3.5 for women) with a higher risk than smoking (ORs 1.9 and 2.5) or drinking (ORs 1.4 and 1.7).

© 2001 Cancer Research Campaign 

BJOC 01-1920 661-667 20/8/01 3:06 pm Page 664

**Table 3** Risk estimates of alcohol habits and dose–response parameters with or without adjustment for chewing and smoking

| Alcohol characteristics | Male | | | | | Female | | | | |
|---|---|---|---|---|---|---|---|---|---|---|
| | Ca/Co | OR (95% CI) | *P* value | Adj OR (95% CI) | *P* value | Ca/Co | OR (95% CI) | *P* value | Adj OR (95% CI) | *P* value |
| Non-alcohol drinker | 189/544 | 1 | | | | 126/276 | 1 | | | |
| Alcohol drinker | 169/172 | 2.8 (0.9–6.3) | 0.085 | 2.2 (0.8–7.5) | 0.15 | 18/12 | 3.3 (1.5–9.5) | 0.04 | 1.3 (0.07–7.6) | 0.06 |
| Frequency (per week) | | | | | | | | | | |
| < 1 | 31/56 | 1.6 (0.06–4.8) | 0.073 | 1.5 (0.05–9.2) | 0.27 | 4//4 | 2.2 (0.8–5.6) | 0.058 | 1.6 (0.05–4.5) | 0.08 |
| 2–4 | 37/55 | 1.9 (0.06–6.3) | 0.065 | 1.4 (0.07–7.2) | 0.23 | 6//5 | 2.6 (0.4–7.3) | 0.052 | 1.5 (0.06–6.2) | 0.08 |
| 5–10 | 63/43 | 4.2 (1.8–10.6) | 0.009 | 2.8 (0.06–8.3) | 0.082 | 8//3 | 5.8 (2.4–11.7) | < 0.001 | 3.6 (0.9–6.3) | 0.006 |
| 10 + | 38/18 | 6.1 (2.7–14.8) | < 0.001 | 4.8 (1.9–11.7) | 0.005 | 0/0 | 0 | 0 | 0 | 0 |
| Duration (years) | | | | | | | | | | |
| <10 | 42/70 | 1.7 (0.7–5.6) | 0.61 | 1.3 (0.08–8.5) | 0.72 | 7//6 | 2.6 (0.4–6.3) | 0.004 | 1.5 (0.09–5.4) | 0.31 |
| 10–19 | 69/76 | 2.6 (0.9–7.2) | 0.04 | 2.1 (0.4–9.3) | 0.08 | 5//4 | 2.7 (0.8–7.8) | 0.002 | 1.3 (0.03–8.4) | 0.53 |
| 20 + | 58/26 | 6.4 (2.6–14.5) | < 0.001 | 5.1 (0.1–7.5) | < 0.001 | 6//2 | 6.6 (3.1–16.3) | < 0.001 | 3.1 (0.2–12.2) | 0.006 |
| Age at start (years) | | | | | | | | | | |
| < 20 | 47/14 | 9.7 (3.6–20.7) | < 0.001 | 7.3 (2.8–16.7) | < 0.001 | 4//1 | 8.8 (3.2–18.5) | < 0.001 | 3.2 (1.4–8.2) | 0.007 |
| 20–29 | 52/56 | 2.7 (0.8–8.3) | 0.002 | 1.8 (0.9–5.4) | 0.075 | 6//4 | 3.3 (1.3–11.6) | 0.006 | 1.7 (0.02–9.4) | 0.46 |
| 30 + | 70/102 | 1.9 (0.8–4.5) | 0.07 | 1.3 (0.3–4.6) | 0.15 | 8//7 | 2.5 (0.9–6.8) | 0.031 | 1.4 (0.03–6.1) | 0.51 |
| Type of alcohol | | | | | | | | | | |
| Non-commercial alcoholic drinks | 63/40 | 4.5 (2.6–6.0) | < 0.001 | 2.4 (0.5–9.6) | 0.007 | 9//5 | 3.9 (1.7–6.8) | 0.003 | 1.9 (0.07–7.5) | 0.09 |
| Process drinks | 52/64 | 2.3 (0.9–4.2) | 0.04 | 1.8 (0.5–6.3) | 0.08 | 5//4 | 2.7 (0.8–5.9) | 0.008 | 1.5 (0.02–9.5) | 0.35 |
| NCAD + PAD | 54/68 | 2.2 (0.7–3.3) | 0.05 | 1.6 (0.4–7.5) | 0.09 | 4//3 | 2.9 (1.6–6.7) | 0.006 | 1.7 (0.06–5.4) | 0.62 |

NCAD = Non-commercial alcoholic drinks; PAD = Process alcoholic drinks.

**Table 4** Risk estimates of different habits of betel nut chewing with additives

| Chewing practices | Male | | | | | Female | | | | |
|---|---|---|---|---|---|---|---|---|---|---|
| | Ca/Co | OR (95% CI) | *P* value | Adj OR (95% CI) | *P* value | Ca/Co | OR (95% CI) | *P* value | Adj OR (95% CI) | *P* value |
| Non-chewer | 30/249 | 1 | | | | 34/153 | 1 | | | |
| Chadha | 68/84 | 6.7 (2.7–16.9) | < 0.001 | 4.9 2.8–11.6 | < 0.001 | 15/8 | 8.4 (2.4–18.8) | <0.001 | 3.4 1.3–5.6 | <0.001 |
| BL + R/G BN | 50/120 | 3.5 (1.3–9.8) | < 0.001 | 1.9 0.08–6.3 | 0.089 | 20/56 | 1.6 (0.9–8.5) | 0.073 | 0.5 0.03–3.7 | 0.422 |
| BL + UG BN | 65/132 | 4.1 (2.2–10.5) | < 0.001 | 2.3 0.7–8.4 | < 0.05 | 15/32 | 2.1 (1.6–10.2) | 0.062 | 0.8 0.06–4.6 | 0.351 |
| BL + R/G BN + D | 40/62 | 5.4 (2.4–15.2) | < 0.001 | 3.1 1.3–6.7 | < 0.01 | 25/14 | 8 (2.2–13.8) | <0.001 | 4.3 1.5–9.7 | <0.001 |
| BL + UG BN + D | 82/54 | 12.6 (5.7–23.8) | < 0.001 | 7.1 3.5–6.7 | < 0.01 | 25/16 | 7 (3.2–17.2) | <0.001 | 3.6 1.4–9.2 | <0.001 |
| BL + S BN + Z | 23/15 | 12.7 (5.8–26.3) | < 0.001 | 6.6 2.8–10.5 | < 0.001 | 10//9 | 5 1.6–11.4 | <0.001 | 2.2 0.4–6.3 | <0.05 |

BL = Betel leaf; R/G = Red/green; UG = Underground; BN = Betel nut; D = Dhapat; S = Supari; Z = Zarda.

 *© 2001 Cancer Research Campaign*

BJOC 01-1920 661-667 20/8/01 3:06 pm Page 665

**Table 5** Risk estimates of practice of spitting, keeping in mouth and swallowing of betel quid after chewing

| Type of chewing | Male | | | | | Female | | | | |
|---|---|---|---|---|---|---|---|---|---|---|
| | Ca/Co | OR (95% CI) | *P* value | Adj OR (95% CI) | *P* value | Ca/Co | OR (95% CI) | *P* value | Adj OR (95% CI) | *P* value |
| Non-chewer | 30/249 | 1 | | | | 34/153 | 1 | | | |
| Spitting | 9//38 | 1.9 (1.2–5.7) | 0.072 | 1.4 (0.06–5.2) | 0.091 | 25/30 | 3.8 (1.5–7.3) | < 0.001 | 1.7 (0.09–5.6) | 0.082 |
| Partially swallow | 34/85 | 3.3 (1.8–9.6) | < 0.001 | 1.6 (0.04–6.2) | 0.167 | 30/46 | 2.9 (1.2–8.6) | < 0.01 | 3.1 (1.2–9.6) | < 0.001 |
| Swallowing | 72/105 | 5.7 (2.3–8.4) | < 0.001 | 3.9 (1.3–9.2) | < 0.001 | 45/50 | 4.1 (2.2–10.6) | < 0.001 | 4.3 (1.9–8.6) | < 0.001 |
| Keeps in mouth | 35/35 | 8.3 (3.2–11.4) | < 0.001 | 5.9 (2.3–11.9) | < 0.001 | 8//7 | 5.1 (2.6–14.2) | < 0.001 | 3.1 (1.2–9.8) | < 0.01 |
| Swallow + Keeps in mouth | 92/80 | 9.5 (3.2–15.9) | < 0.001 | 6.3 (1.4–13.2) | < 0.001 | 2//2 | 4.5 (1.6–9.2) | < 0.001 | 2.9 (1.6–7.4) | < 0.01 |

**Table 6** Risk estimates of different combinations of betel nut chewing and smoking (adjusted for alcohol)

| | Male | | | | | Female | | | | |
|---|---|---|---|---|---|---|---|---|---|---|
| | Ca/Co | OR (95% CI) | *P* value | Adj OR (95% CI) | *P* value | Ca/Co | OR (95% CI) | *P* value | Adj OR (95% CI) | *P* value |
| NCh & NSm | 26/227 | 1 | | | | 31/142 | 1 | | | |
| Chadha | 20/39 | 4.5 (2.7–8.3) | 0.003 | 3.2 (1.6–9.5) | 0.004 | 8//5 | 7.3 (2.4–13.5) | < 0.001 | 6.2 (2.4–12.1) | < 0.001 |
| Chadha+BSm | 12//17 | 6.2 (2.6–10.4) | 0.001 | 5.7 (1.8–10.3) | 0.01 | 4//3 | 6.1 (3.2–12.9) | < 0.001 | 5.1 (1.9–10.3) | < 0.01 |
| Chadha+CSm | 11//19 | 5.1 (2.4–9.8) | 0.001 | 4.3 (2.1–9.6) | 0.003 | 3//3 | 4.6 (2.7–10.3) | 0.004 | 3.7 (1.8–6.5) | 0.006 |
| BL+R/GBN | 22/63 | 3 (1.5–7.2) | 0.02 | 2.4 (1.2–5.5) | 0.09 | 12//40 | 1.4 (0.4–5.6) | 0.4 | 0.5 (0.01–4.3) | 0.52 |
| BL+R/GBN+BSm | 20/35 | 5 (2.3–10.6) | < 0.001 | 4.3 (2.6–8.3) | 0.01 | 6//10 | 2.7 (1.3–7.5) | 0.07 | 1.4 (0.02–5.2) | 0.41 |
| BL+R/GBN+CSm | 14/30 | 4.1 (1.8–10.8) | < 0.001 | 3.2 (1.8–6.7) | 0.005 | 4//10 | 1.8 (0.6–6.3) | 0.5 | 0.8 (0.06–3.8) | 0.66 |
| BL+UGBN | 34/68 | 4.4 (1.8–9.3) | 0.002 | 2.6 (1.4–6.5) | 0.008 | 10//26 | 1.8 (0.3–4.5) | 0.31 | 1.2 (0.05–4.6) | 0.48 |
| BL+UGBN+BSm | 20/34 | 5.1 (2.1–10.5) | < 0.001 | 4.3 (2.3–9.8) | 0.007 | 3//5 | 2.7 (1.6–7.6) | 0.15 | 1.9 (0.2–5.7) | 0.26 |
| BL+UGBN+CSm | 19/37 | 4.5 (2.3–8.6) | < 0.001 | 3.8 (1.7–10.5) | 0.006 | 2//4 | 2.3 (1.5–9.6) | 0.21 | 1.5 (0.3–7.6) | 0.37 |
| BL+R/GBN+D | 20/32 | 5.5 (1.6–9.8) | < 0.001 | 4.8 (2.6–10.3) | < 0.001 | 16/15 | 4.9 (2.5–9.6) | < 0.001 | 3.8 (1.3–8.5) | 0.004 |
| BL+R/GBN+D+BSm | 17/20 | 7.4 (2.1–11.3) | < 0.001 | 6.5 (2.8–11.6) | < 0.001 | 7//3 | 10.7 (3.3–13.7) | < 0.001 | 8.5 (2.6–16.3) | < 0.001 |
| BL+R/GBN+D+CSm | 12//19 | 5.5 (1.3–10.4) | < 0.001 | 5 (1.8–10.8) | < 0.001 | 3//2 | 6.9 (2.8–12.6) | < 0.001 | 4.5 (1.6–8.4) | < 0.001 |
| BL+UGBN+D | 35/20 | 15.3 (7.1–23.8) | < 0.001 | 9.5 (3.3–20.8) | < 0.001 | 12//6 | 9.2 (3.6–15.4) | < 0.001 | 6.6 (2.4–11.5) | < 0.001 |
| BL+UGBN+D+BSm | 26/9 | 25.2 (10.3–31.2) | < 0.001 | 15.3 (4.6–28.7) | 0.003 | 8//1 | 36.6 (18.5–48.6) | < 0.001 | 27.4 (14.3–41.5) | < 0.001 |
| BL+UGBN+D+CSm | 25/14 | 15.6 (6.3–21.2) | < 0.001 | 5.1 (2.4–17.6) | 0.006 | 5//1 | 22.9 (7.5–42.7) | < 0.001 | 16.1 (8.1–27.3) | < 0.001 |
| BL+SBN+Z | 12//15 | 7 (2.6–13.3) | < 0.001 | 5.6 (2.3–10.3) | < 0.001 | 5//7 | 3.3 (1.7–8.6) | 0.03 | 1.9 (0.4–6.5) | 0.28 |
| BL+SBN+Z+BSm | 6//8 | 6.5 (2.7–12.2) | < 0.001 | 4.1 (1.8–9.7) | 0.005 | 3//3 | 4.6 (2.3–10.5) | 0.02 | 2.8 (1.3–7.6) | 0.09 |
| BL+SBN+Z+CSm | 7//10 | 6.1 (2.3–13.5) | <0.001 | 3.7 (1.4–7.6) | 0.02 | 2//2 | 4.5 (1.9–12.6) | 0.005 | 2.4 (1.1–9.4) | 0.04 |

NCh = Non chewer; NSm = Non smoker; BSm = Bidi smoker; CSm = Cigarette smoker; BL = Betel leaf; BN = Betel nut; R/G = Raw/Green; UG = Underground; D = Dhapat; Z = Zarda.

© 2001 Cancer Research Campaign

BJOC 01-1920 661-667 20/8/01 3:06 pm Page 666

**Table 7** Risk estimates of different combinations of betel nut chewing and alcohol drinking (adjusted for smoking)

| | Male | | | | | Female | | | | |
|---|---|---|---|---|---|---|---|---|---|---|
| | Ca/Co | OR (95% CI) | *P* value | Adj OR (95% CI) | *P* value | Ca/Co | OR (95% CI) | *P* value | Adj OR (95% CI) | *P* value |
| NCh & NAD | 22/218 | 1 | | | | 28/149 | 1 | | | |
| Chadha | 16/35 | 4.5 (1.6–10.3) | 0.003 | 3.8 (1.9–8.5) | 0.003 | 7//6 | 6.2 (2.4–15.9) | <0.001 | 5.8 (2.1–12.4) | < 0.001 |
| Chadha+NCAD | 19/28 | 6.7 (2.8–14.4) | < 0.001 | 6.1 (2.6–12.8) | 0.004 | 4//3 | 7.1 (2.8–19.7) | < 0.001 | 6.3 (2.4–14.3) | < 0.001 |
| Chadha+PA | 15/23 | 6.5 (2.4–15.2) | < 0.001 | 5.3 (2.2–13.1) | 0.002 | 3//3 | 5.3 (1.5–16.3) | < 0.001 | 4.4 (1.7–9.5) | < 0.001 |
| BL+R/GBN | 21/53 | 3.9 (1.4–12.6) | 0.008 | 2.8 (1.3–7.5) | 0.06 | 10//27 | 2 (0.7–8.5) | 0.04 | 1.4 (0.3–6.5) | 0.24 |
| BL+R/GBN+NCAD | 26/41 | 6.3 (2.5–11.4) | 0.002 | 5.6 (2.2–9.6) | 0.004 | 7//20 | 2 (0.3–10.6) | 0.08 | 1.6 (0.2–9.3) | 0.41 |
| BL +R/GBN+PA | 14/25 | 5.5 (1.9–10.8) | < 0.001 | 4.2 (1.8–10.5) | < 0.001 | 6//12 | 2.7 (1.1–9.5) | < 0.01 | 1.7 (0.6–8.5) | 0.15 |
| BL+UGBN | 22/58 | 3.8 (1.2–9.6) | 0.004 | 3.1 (1.6–8.5) | 0.002 | 9//15 | 3.2 (1.8–11.5) | < 0.001 | 2.4 (0.9–7.2) | < 0.01 |
| BL+UGBN+NCAD | 39/46 | 8.4 (3.4–14.5) | < 0.001 | 6.2 (2.4–11.3) | < 0.001 | 7//10 | 3.7 (1.3–10.7) | < 0.001 | 2.1 (1.3–5.4) | 0.04 |
| BL+UGBN+PA | 20/41 | 4.8 (1.6–11.2) | 0.001 | 3.6 (1.7–9.5) | < 0.001 | 5//9 | 3 (1.5–8.6) | < 0.001 | 1.9 (0.4–7.5) | 0.09 |
| BL+R/GBN+D | 21/38 | 5.5 (2.3–11.8) | < 0.001 | 5 (1.7–10.6) | < 0.001 | 8//8 | 5.3 (1.7–10.8) | < 0.001 | 4.2 (1.6–10.5) | < 0.001 |
| BL+R/GBN+D+ NCAD | 20/26 | 7.6 (2.8–12.3) | < 0.001 | 7.3 (2.5–12.8) | < 0.001 | 7//6 | 6.2 (2.3–14.2) | < 0.001 | 5.6 (2.3–12.4) | < 0.001 |
| BL+R/GBN+D+PA | 12/22 | 5.4 (1.8–10.6) | < 0.001 | 4.8 (1.7–9.3) | < 0.001 | 5//3 | 8.9 (2.4–19.8) | < 0.001 | 7.3 (2.6–10.3) | < 0.001 |
| BL+UGBN+D | 26/20 | 12.9 (3.2–18.5) | < 0.001 | 10.3 (3.6–20.8) | < 0.001 | 12//5 | 12.8 (4.2–20.8) | < 0.001 | 10.4 (2.6–18.5) | < 0.001 |
| BL+UGBN+D+ NCAD | 31/14 | 21.9 (7.5–32.4) | < 0.001 | 18.5 (5.6–27.3) | < 0.001 | 9//3 | 16 (8.3–26.4) | < 0.001 | 13.5 (3.1–20.6) | < 0.001 |
| BL+UGBN+D+PA | 12//15 | 7.9 (1.9–14.5) | < 0.001 | 6.3 (2.5–14.7) | < 0.001 | 5//2 | 13.3 (5.4–21.6) | < 0.001 | 10.6 (3.2–18.2) | < 0.001 |
| BL+SBN+Z | 9//6 | 14.9 (4.6–22.8) | < 0.001 | 8.4 (2.6–17.5) | < 0.001 | 7//4 | 9.3 (3.6–18.5) | < 0.001 | 8.4 (3.1–16.3) | < 0.001 |
| BL+SBN+Z+NCAD | 6//3 | 19.8 (5.3–28.6) | < 0.001 | 12.1 (4.3–21.4) | < 0.001 | 3//2 | 8 (2.4–17.3) | < 0.001 | 6.5 (2.7–14.6) | < 0.001 |
| BL+SBN+Z+PA | 7//4 | 17.3 (4.2–24.5) | < 0.001 | 13.6 (4.6–22.5) | < 0.001 | 2//1 | 10.6 (3.5–20.4) | < 0.001 | 7.3 (1.8–15.3) | < 0.001 |

NC = Non chewer; NAD = Non alcohol drinker; BL = Betel leaf; BN = Betel nut; R/G = Raw/Green; UG = Underground; D = Dhapat; Z = Zarda; NCAD = Non-commercial alcoholic drinks (local beverages = chulai, rice beer, high spirited country liquor etc.); PA = Process alcohol (foreign beverages = whisky, rum, brandy, beer wine etc.).

### DISCUSSION

Betel nut chewing with or without tobacco has been shown to be independently associated with the development of oesophageal cancer in Assam and there are clear dose-related responses that indicate a causal effect. Risks are higher for men than for women and further evidence from the data shows that male chewers start the habit at a younger age, use tobacco more often and chew both more frequently during the day and for longer periods of time. Similar findings have also been reported from elsewhere in India (Jussawalla, 1971, 1981). However, in Assam it has been found that the risk from chewing betel nut and tobacco together is higher than that from betel nut alone and this differs from the earlier findings from Bombay where chewing betel nut alone gave a substantially higher risk, apparently because the juices from the quid with tobacco were usually spat out while those from betel nut alone were habitually swallowed (Jussawalla, 1971).

The betel nut (*Areca catechu* L) has been shown to have carcinogenic potential (Suri et al, 1971; Sharan and Wary, 1992) and 3-methyl nitrosamine propionitrile (MNPN), a potent carcinogen (Nair et al, 1987) and safrole-like DNA adducts (Chen et al, 1999) have been detected in the saliva of betel chewers. Both saliva and the active alkaloid, arecoline, present in the nut have been shown to be genotoxic and mutagenic (Chetia et al, 1996; Chaterjee and Deb, 1999; Mahanta et al, 1999; Saikia et al, 1999). Contamination of areca nuts has also been found by fungi such as *Aspergillus flavus*, *A. niger* and *Rhisopus* sp. (Bandre, 1983; Borle and Gupta, 1987) which can produce carcinogenic *aflatoxins*.

Clearly the effect of chewing is greatest on the buccal mucosa and many studies have indicated a strong dose–response relationship with tumours of the oral cavity (Blot et al, 1997). However, components of the betel quid are absorbed through the mucous membrane by chewers while some portion is also swallowed so that the oesophagus is also affected. The present study strongly indicates that betel nut chewing is probably the most important risk factor for oesophageal cancer in Assam and shows the need yet again for public education to highlight the risks associated with this deeply entrenched local habit.

 *© 2001 Cancer Research Campaign*

**Table 8** Risk factors for cancer oesophagus related to isolated and combined habits

| Habits | Male | | | CF | EF | Female | | | CF | EF |
|---|---|---|---|---|---|---|---|---|---|---|
| | Ca/Co | RR (95% CI) | *P* value | | | Ca/Co | RR (95% CI) | *P* value | | |
| No habit | 32/217 | 1 | | | | 26/132 | 1 | | | |
| Chew only | 67/133 | 3.4 (1.2–9.5) | 0.005 | 0.18 | 0.7 | 78/113 | 3.5 (1.4–10.3) | 0.004 | 0.54 | 0.71 |
| Smoke only | 27/98 | 1.9 (0.3–5.6) | 0.23 | 0.08 | 0.47 | 5//10 | 2.5 (0.8–7.3) | 0.08 | 0.03 | 0.61 |
| Drink only | 22/106 | 1.4 (0.1–4.5) | 0.46 | 0.06 | 0.29 | 4//12 | 1.7 (0.5–5.8) | 0.63 | 0.03 | 0.41 |
| Chew + Smoke | 83/67 | 8.4 (2.6–14.3) | < 0.001 | 0.23 | 0.88 | 8//5 | 8.1 (2.3–12.9) | < 0.001 | 0.06 | 0.87 |
| Smoke + Drink | 28/27 | 7 (2.1–13.4) | < 0.001 | 0.08 | 0.86 | 4//5 | 4.1 (1.3–10.3) | 0.002 | 0.03 | 0.75 |
| Alcohol + Chew | 25/31 | 5.5 (1.9–14.3) | < 0.001 | 0.07 | 0.82 | 12//8 | 7.6 (2.1–16.3) | < 0.001 | 0.08 | 0.86 |
| Chew + Drink + Smoke | 74/37 | 13.6 (4.5–21.3) | < 0.001 | 0.21 | 0.93 | 7//3 | 11.8 (3.7–21.5) | <0.001 | 0.05 | 0.92 |

Chew = Chew betel nut with or without tobacco; Drink = Drinks alcohol of any form; Ca = Cases; Co = Control; RR = Relative risks; CF = Case fraction (Proportion of all cases in ith category of exposure); EF = Aetiological fraction (EFi = RRi-1/RRi where i is category exposure group).

### ACKNOWLEDGEMENTS

We thank Dr GG Ahmed, Director, Dr Bhubaneswar Barooah Cancer Institute, Guwahati. Assam. India for permitting us to do our study in his institute. Our sincere thanks to Dr A Nanda Kumar, Dy Director General (Sr Grade). National Cancer Registry Programme (ICMR). Kidwai Memorial Institute of Oncology. Bangalore, India for his kind permission to analyse our work. We also acknowledge the technical help of Mr Muralidhar. Senior Investigator. NCRP (ICMR). Kidwai Memerial Institute of Oncology, Bangalore, during the analysis of our work.

### REFERENCES

Bandre TR (1983) *Toxicity studies of food contaminants*. (Ph.D. Thesis). Nagpur University, Nagpur (India)

Bhansle RB, Murti PR, Daftary DK and Mehta FS (1979) An oral lesion in tobacco lime users in Maharashtra, India. *Ind J Oral Pathol* **8**: 47–52

Blot WJ, McLaughlin JK, Devesa SS and Fraument J Jr (1997) Cancer of oral cavity and pharynx. In: *Cancer Epidemiology and Prevention* (D Schottenfeld, JG Searle and J Fraumeni Jr eds) Oxford University Press: London

Borle RM and Gupta DS (1987) Fungal contamination of Arecanut. *Indian J Pathol Microbiol* **30**: 357–360

Breslow NE and Day NE (1980) *The analysis of case–control studies. Statistical methods in cancer research*, Vol. 1, No. 32, IARC scientific publications

Chatterjee A and Deb S (1999) Genotoxic effect of arecoline given either by the peritoneal or oral route in murine bone marrow cells and the influence of N-acetylcysteine. *Cancer-Lett* **139**: 23–31

Chen CL, Chi CW, Chang KW and Liu TY (1999) Safrole-like DNA adducts in oral tissue from oral cancer patients with a betel quid chewing history. *Carcinogenesis* **20**: 2331–2334

Chetia M, Mahanta J and Sharma SK (1996) Toxic effect of tender and fermented ripe betel nuts on root growth and root tip cells of Allium cepa. *J Environ Biol* **17**: 251–256

Jussawalla DJ (1971) Epidemiological assessment of aetiology of oesophageal cancer in Greater Bombay. In: *Monograph No.1 International seminar on epidemiology of oesophageal cancer*; Bangalore, India, 4 November, 20–30

Jussawalla DJ (1981) Oesophageal cancer in India. *J Cancer Res Clin Oncol* **99**: 29–33

Jussawalla DJ and Deshpande VA (1971) Evaluation of cancer risk in tobacco chewers and smokers: an epidemiologic assessment. *Cancer* **28**: 244–252

Kinjo Y, Cui Y, Akiba S, Watanabe S, Yamaguchi N, Sobue T, Mizuno S and Beral V (1998) Mortality risks of oesophageal cancer associated with hot tea, alcohol, tobacco and diet in Japan. *J Epidemiol* **8(4)**: 235–243

Levin ML (1953) The occurrence of Lung cancer in Man. Acta Unio Int. *Contra Cancrum* **9**: 531–541

Mahanta J, Chetia M and Chelleng PK (1999) The toxic properties of saliva of betel nut chewers on a plant test system (*Allium cepa* L) *J Environ Biol* **20**: 85–88

Munoz N and Day NE (1997) Esophageal Cancer: *Cancer Epidemiology and Prevention*: D Scottenfeld, JG Searle and J Fraumeni. Oxford University Press, London

Nair J, Nair UJ, Oshima H, Bhide SV and Bartsh H (1987) *Endogenous nitrosation in the oral cavity of chewers while chewing betel quid with or without tobacco*. IARC Scientific Publications, **84**: 465–469

Nandakumar A, Anantha N, Pattabhiraman V, Probhakaran PS, Dhar M, Puttaswamy K, Venugopal TC, Reddy NMS, Rajanna, Vinutha AT and Srinivas (1996) Importance of anatomical subsite in correlating risk factors in cancer of the oesophagus-report of a case-control study. *Br J Cancer* **73**: 1306–1311

National Cancer Registry Programme (NCRP) of India (1984–1989) *Annual Reports*, Indian Council of Medical Research, New Delhi

Saikia JR, Schneeweiss FH and Sharan RN (1999) Arecoline-induced changes of poly-ADP-ribosylation of cellular proteins and its influence on chromatin organization *Cancer-Lett* **139**: 59–65

Sharan RN and Wary KK (1992) Study of unscheduled DNA synthesis following exposure of human cells to arecoline and extracts of betel nut in vitro. *Mutation Research* **278**: 271–276

Suri K, Goldman HM and Wells H (1971) Carcinogenic effects of DMSO extracts of betel nut on hamster check pouch. *Nature* (London), **230**: 383

Tuyns AJ, Pequignot G and Jensen OM (1977) Oesophageal cancer in Ille et Villaine in relation to alcohol and tobacco consumption. Multiplicative risk. *Bull Cancer* **64**: 45–50

*© 2001 Cancer Research Campaign*
